# Allograft Model of Aortic Arch Segment Grafting to Abdominal Aorta Through End-to-Side Anastomosis in Mice

**DOI:** 10.1007/s12265-024-10495-w

**Published:** 2024-02-26

**Authors:** Chiyu Liu, Qi Chen, Mingyuan He, Yulin Liao

**Affiliations:** 1 Department of Cardiology, State Key Laboratory of Organ Failure Research, Guangdong Provincial Key Laboratory of Heart Function and Microcirculation, Nanfang Hospital, Southern Medical University, 1838 Guangzhou Avenue North, Guangzhou, 510515 Guangdong Province China; 2 Cardiovascular Center, the Affiliated Sixth Hospital, School of Medicine, South China University of Technology, Foshan, China

**Keywords:** Aortic arch transplantation,, ApoE gene knockout mice,, Mouse model,, Regression of atherosclerosis,, Syngeneic heterotopic transplantation

## Abstract

The mouse aortic transplantation model is a valuable tool for investigating the mechanisms of atherosclerosis regression, but few laboratories can generate it due to the operation difficulty, especially for the style of end-to-side anastomosis, which facilitates syngeneic heterotopic transplanting a plaque-rich aortic arch into the abdominal aorta. Here we provide a modified protocol for generating this allograft model, which is capable of overcoming several critical surgical challenges such as separating a longer abdominal aorta segment, reducing bleeding and thrombosis, optimizing aortotomy, and improving end-to-side anastomosis to guarantee a potent graft. By transplanting plaque-rich aortic arches into the abdominal aorta of wildtype mice, a high operation success rate (over 90%) was noted with aortic clamping time under 60 min, the graft potency was satisfactory evidenced by examinations of micro-CT, ultrasound, and lower limb blood flow measurement, while a significant atherosclerosis regression was observed in the grafts at 1 week after transplantation.

**Graphical Abstract:**

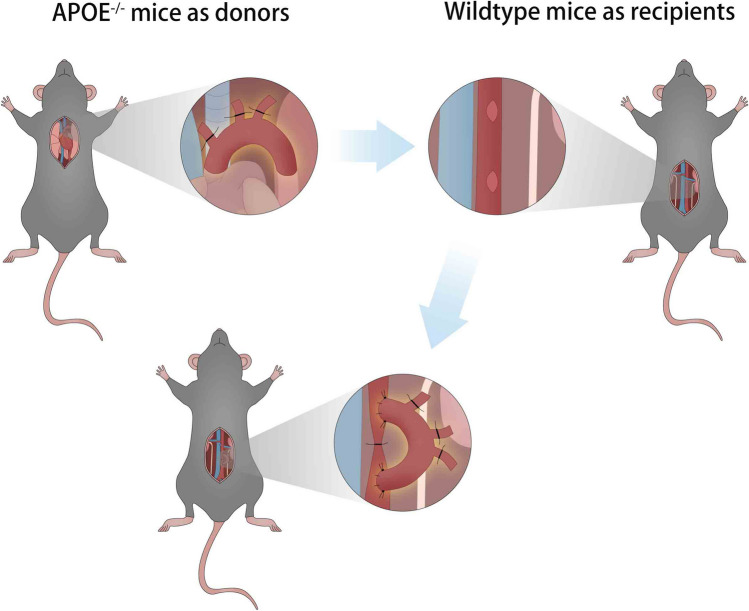

## Background

The mouse aortic transplantation model has been introduced to study the plaque regression [1] and allograft arteriopathy [2] for many years, but the technical difficulties of small vessel anastomoses largely limit its widespread use. Plaque regression is an interesting topic and the aortic allograft model in mice would be very helpful for clarifying the potential mechanisms of plaque regression. Syngeneic heterotopic allograft has been recommended, which is created by transplanting a segment of descending thoracic or abdominal aorta or aortic arch containing atherosclerotic lesions from a hypercholesterolemic mouse into the abdominal aorta of a normocholesterolemic recipient [1, 3]. Although the heterotopic transplantation of the aortic arch model was reported 20 years ago, few laboratories are good at using this model to investigate plaque regression.

It is relatively easy to transplant a donor thoracic aorta or abdominal aorta into the recipient abdominal aorta through end-to-end anastomosis. Considering that the aortic arch develops much more significant atherosclerotic plaque than the thoracic and abdominal aorta, transplanting the aortic arch is a better choice for the study of plaque regression [1, 4]. Due to the curved shape and larger diameter of the aortic arch, end-to-side anastomosis is recommended for aortic arch transplantation into the abdominal aorta, but the technique difficulty is larger than end-to-end anastomosis.

The aortic arch transplantation procedure faces several challenges that largely affect the operation success rate. First, a longer abdominal aorta segment needs to be exposed and separated from the inferior vena cava (IVC) in order to meet the requirements of end-to-side anastomosis (particular attention should be paid to the separation skills to avoid the rupture of the fragile arterio-venous walls and massive bleeding). Second, appropriate proximal and distal abdominal aortotomies are necessary to ensure an unobstructed graft. Finally, improvement of end-to-side anastomosis procedure to avoid the direct closure of aortic incision or the graft ends. Here we describe in detail a reproducible aortic arch transplantation procedure as well as the representative results of operation and plaque regression.

## Materials and Methods

All animal procedures in this study were conducted in accordance with the Guide for the Care and Use of Laboratory Animals published by the US National Institutes of Health (NIH Publication No. 85–23, revised in 1996) and approved by the Animal Ethics Committee of Nanfang Hospital, Southern Medical University (approval number: IACUC-LAC-20220830–001). The wildtype C57BL/6 J mice were purchased from the Animal Center of Southern Medical University and the ApoE^−/−^ C57BL/6 J mice from Beijing Vital River Laboratory Animal Technology Co., Ltd. All mice were raised in the specified pathogen free (SPF) space that offers a 12/12 h dark/light cycle and sufficient food and water. All mice were 4 weeks old and weighed 13 ~ 15 g when introduced in the facility. The quarantine time was 2 weeks. For the atherosclerosis regression study, ApoE^−/−^ mice (8 weeks of age and weight 22 ~ 24 g) were fed with Western diet (containing 21% fat, 0.15% cholesterol; Guangdong Medical Laboratory Animal Center) for 16 weeks prior to transplantation, while the age-matched wildtype mice were fed with chow diet.

### Preoperative Preparation

- 1. Autoclave all surgical instruments before operation (see Table 1).

**Table 1:** List of surgical instruments

Name	Company	Catalog number	Specifications
Hemostatic forceps	R&S	RS-Z100	Length: 12.5 cm
“Vannas” micro-scissors	ELGBIO	18–0000	Length: 8.5 cm; tip: straight
Micro-forceps	ELGBIO	18–1050	Length: 11 cm; tip: curved, 0.1 × 0.06 mm
Micro-forceps	ELGBIO	18–1000	Length: 11 cm; tip: straight, 0.05 × 0.02 mm
Elbow ophthalmic tweezer	Jinzhong	JD1060	
Ophthalmic scissors	Jinzhong	Y0030	
Micro-retractor	XB-BIO	XB-4–50	
Micro-needle forceps	FeiBo	EMT-140-W	Length: 14 cm; tip: curved
Micro-forceps	ELGBIO	18–1071	Length: 13 cm; tip: curved, 0.15 mm
Micro-forceps	ELGBIO	18–1070	Length: 13 cm; tip: straight, 0.15 mm
11–0 nylon suture	CONPUVON	DYZ-11–0	Shape of needle: round; camber of needle: 3/8; length of needle diameter: 4 mm; thickness of needle diameter: 0.7 mm; thickness of suture: 11–0
10–0 nylon suture	CONPUVON	DYZ-10–0	Shape of needle: round; camber of needle: 3/8; length of needle diameter: 5 mm; thickness of needle diameter: 1 mm; thickness of suture: 10–0
8–0 nylon suture	CONPUVON	DYZ-8–0	Shape of needle: round; camber of needle: 3/8; length of needle diameter: 6 mm; thickness of needle diameter: 1.5 mm; thickness of suture: 8–0
5–0 nylon suture	CONPUVON	DYZ-5–0	Shape of needle: round; camber of needle: 3/8; length of needle diameter: 8 mm; thickness of needle diameter: 2.5 mm; thickness of suture: 5–0
5–0 silk suture	SKJYLEAN	F34004-50	Length of suture: 60 cm; thickness of suture: 5–0

- 2. Set up the stereo microscope and adjust the magnification to 20 × –40 × .
- 3. Place the operating table under the microscope.
- 4. Pre-heat a 500 mL bottle of saline at 37 °C. Add 2 mL of heparin sodium (containing 12,500 units) into 125 mL of normal saline to prepare 100 U/mL heparinized saline and pre-cooled at 4 °C.
- 5. Adjust the heating blanket to 37 °C for postoperative rewarming of the recipient mouse.

### Donor Procedure

1. Anesthetize the donor mouse (we used ApoE^−/−^ mouse fed with high-fat diet) by intraperitoneal injection of 50 mg/kg pentobarbital and assess the depth of anesthesia by pinching the mouse’s feet to ensure the disappearance of foot retraction reaction. Apply analgesia to the mouse by subcutaneous injection of 5 mg/kg carprofen before the operation.
2. Once the narcosis is observed, remove the eyeball with an elbow ophthalmic tweezer. Collect the gushing blood with a 1.5 mL EP tube (at least 1 mL blood) for follow-up testing of lipid levels, etc.
3. Fix the mouse on the operating table in a supine position with extended limbs and spray the entire chest with alcohol to prevent body hair from sticking to the instruments.
4. Cut an incision under the xiphoid process of the sternum and take this as a starting point to cut the chest wall on both sides. Fully expose the chest organs and make the thymus be clearly visible.
5. Press the heart with micro-forceps, make a cut in the right atrium and inject 8 mL heparinized saline slowly into the left ventricle for more than 3 min (a 10 mL syringe with a 25G needle is recommended).
  Note: Clear fluid discharge from the right atrium or whitening of the liver is a sign of good perfusion.
6. Carefully cut the tissue around the aortic arch using “Vannas” micro-scissors and micro-forceps, and then fully expose the aortic arch and its three branches.
  Tips: First cut out the thymus and the left lung to get the outline of the aortic arch. Hold the connective tissue with micro-forceps and gently pull. When obvious demarcation comes out between the aortic arch and the connective tissue, cut down the connective tissue along the demarcation line to prevent accidental injury to the aortic arch.
7. Ligate the three branches of the aortic arch (brachiocephalic trunk, left common carotid artery, and left subclavian artery) at the bifurcations with 8–0 nylon sutures (either surgical or square knot is workable, two knots are recommended for ligating a branch because plaques are largely distributed at bifurcations).
  Note: When trimming the thread, leave 1–2 mm length of suture for subsequent clip of the aortic arch.
8. Transect the aortic arch 2 mm proximal to the brachiocephalic trunk and 3 mm distal from the left subclavian artery. Transect the three branches 1 mm away from the knot.
9. Immerse the aortic arch graft in heparinized saline at 4 °C.

### Recipient Procedure

1. Anesthetize the mouse (C57 BL/6 J wildtype mouse fed with chow diet) by intraperitoneal injection of 50 mg/kg pentobarbital and assess the depth of anesthesia by pinching the mouse’s feet to ensure the disappearance of foot retraction reaction. Apply analgesia to the mouse by subcutaneous injection of 5 mg/kg carprofen before the operation.
  Note: Intraperitoneal injection of anesthetics into the recipient can be performed prior to the donor procedure. When the donor procedure is finished, adequate anesthesia of the recipient is usually reached. Recommend evaluating the depth of anesthesia every 15 min during the recipient procedure. If the recipient has reactions to the stimulus, supplement with 1/4 to 1/3 original dose of pentobarbital.
2. Fix the mouse incisors with 5–0 nylon suture and the fully extended limbs with tapes to the operating table in a supine position. Apply the depilatory paste evenly to the abdominal skin below the xiphoid process and above the pubis and wipe up the removed hair with sterile cotton balls. Disinfect the abdomen skin with iodophor cotton balls three times.
3. Make a median abdominal incision on the recipient mouse, then drag abdominal contents out with sterile cotton swabs and open the abdominal wall with a micro-retractor (the dark red IVC is clearly visible to the naked eye). Wrap the abdominal contents with sterile gauze moistened with warm saline solution. Moisten the exposed tissue with warm saline solution intermittently during surgery.
4. Separate the infrarenal aorta from the IVC carefully under a microscope by using micro-forceps until expose a 7 mm segment or more of the abdominal aorta between the origin of the left renal artery and the bifurcation of the abdominal aorta. Use 8–0 nylon sutures to ligate the collateral vessels of this segment (there are usually 3–5 collateral vessels in each mouse with only small variations among individual mice in the number and location of collateral vessels).
  Tips: Improper operation in this step would be high likely to cause bleeding of IVC and abdominal aorta. It is advisable to begin the separation step at the origin of the left renal vessels. Carefully tear the fatty tissue on the surface of the aorta and the IVC to expose the gap between the aorta and vein. The abdominal aorta is not closely connected to the IVC adjacent to the origin of the left renal vessels, so a distinct gap between them will appear after a simple tearing. Then, starting with the exposed gap, continue to tear the distal adipose tissue to expose more gap and collateral vessels. When it comes to segments that are difficult to separate (for example, the segment between the lumbar artery and the bifurcation of the abdominal aorta), it is useful to bluntly dissect the transparent connective tissue between the aorta and vein firstly with micro-forceps (0.1 mm thick at the tip), then loosen the junction between IVC and abdominal aorta by adding a small amount of saline solution when the connective tissue on the surface of aorta is pulled by micro-forceps.
5. Thread 5–0 silk sutures at both ends of the separated abdominal aortic segment and 8–0 nylon suture in the middle of the segment.
  Note: The proximal end usually requires two silk stitches due to the high blood pressure, and the distal and middle only need one.
6. Tie the silk sutures at the distal end and the nylon suture in the middle consecutively, leaving the proximal silk suture unknotted. Puncture aortic lumen 0.5 mm proximal to the knot in the middle with the needle attained to a 10–0 nylon suture, then pass the needle through the lumen along the aortic longitudinal axis and the distance between the exit point and the entry point is 1/8 of the needle length. Gently lift the needle upwardly and cut out a section of the anterior aortic wall along the path of the needle in the lumen with “Vannas” micro-scissors, creating an approximate 1 mm elliptical longitudinal opening with the trim edge (referred to as distal incision of the abdominal aorta). Flush the remaining blood out of the lumen with ice-heparinized saline.
7. Take out the aortic arch graft and align its distal opening with the distal incision of the abdominal aorta. Place the stay sutures at 6 and 12 o’clock positions by using 11–0 nylon suture. Tie a square knot at 12 o’clock but only thread without a knot at 6 o’clock for the time being. Perform continuous sutures from 12 o’clock to 6 o’clock with running sutures (11–0 nylon suture). First anastomose the left cut edge in 3 stitches, followed by tying the stay suture at 6 o’clock and tying the running suture to the stay suture at 6 o’clock with a surgical knot. Rotate the operating Table 180° (the positions of two stay sutures are reversed) and continue to perform continuous suture on the right cut edge. Before the final knot tied with stay sutures and running sutures, flush the anastomosis again with heparinized saline to get rid of residual blood in the abdominal aorta and the graft.
  Note: When anastomose the left or right cut edge, do not hurry to pull the thread tight after each stitch is finished. Until the third stitch is finished, then pull all the threads tight.
8. Rotate the operating table 180° to return to the original position and tie the silk sutures at the proximal end and make a longitudinal opening (referred to as proximal incision of the abdominal aorta) the same as the distal end. Then perform end-to-side anastomosis on the proximal end in the distal manner.
9. Loosen the distal knot to allow the retrograde flow into the aortic arch graft, and then stop the bleeding with sterile cotton swabs.
10. After obtaining satisfactory hemostasis, loosen the first proximal knot. Then slowly loosen the second knot, and the blood will flood into the graft, causing a large amount of blood seepage at the anastomosis. At this time, quickly hold the cotton swabs against the anastomosis for 5 min.
  Note: During hemostasis, it is not recommended to heavily press the anastomosis and graft, as it may promote thrombosis. Successful hemostasis is judged by no bleeding within 5 min after withdrawing the cotton swabs.
11. Graft pulsation is a sign of patency of the proximal anastomosis. If a significantly reduced diameter and dark red blood appear in the distal abdominal aorta, it usually indicates the distal anastomosis is obstructed.
12. Flush the abdominal cavity with heparinized saline and wait for absorption. Return the abdominal contents and close the muscle and skin layers with 5–0 nylon sutures. Disinfect the abdomen skin with iodophor cotton balls three times.
13. Place the mouse on the heating blanket in a separate cage to promote recovery from anesthesia. Assess the sensory and motor function of the hind limbs after mouse resuscitation on the following day. Give buprenorphine via the drinking water for the next 2 days.

### In Vivo Micro-CT Imaging

Note: Imaging of the aortic arch graft is taken by micro-CT with a Super Nova Micro-CT device (PINGSENG, Jiangsu, China) at 35 µm resolution, which is carried out as described before [5].

- 1. Anesthetize the mouse with 3% isoflurane via inhalation.
- 2. Inject the mouse with a dose of 300 µl/25 g iodine contrast medium via a tail vein catheter.
- 3. Place the mouse in a supine position on the rotation center, aligned with the center axis of the animal bed.
- 4. Adjust the isoflurane concentration in the anesthetic nose cone between 1.5 and 2.5%.
- 5. Use ReconDaemon and Cruiser software for data acquisition during scanning. Reconstruct two-dimensional grayscale image slices along the coronal, sagittal, and vertical axis using Avatar 3 software.

### In Vivo Ultrasound Imaging

Note: Imaging of the aortic arch graft is obtained by a high-frequency ultrasound (VisualSonics, Toronto, Canada) as previously described [6–8].

1. Anesthetize the mouse with 3% isoflurane via inhalation.
2. Place the mouse in a supine position on the heating platform and tape its claws to the electrode to obtain an ECG recording through a system attached to the ultrasonic machine.
3. Adjust the isoflurane concentration in the anesthetic nose cone between 1.5 and 2.5% to maintain the heart rate of the mouse between 450 and 550 beats/min.
4. Remove the abdominal hair of the mouse with depilatory cream and apply ultrasonic gel to the abdominal skin.
5. Rotate the platform and adjust the y-axis and x-axis knobs for optimal imaging of the longitudinal native abdominal aorta and the implanted aortic arch.
6. Measure the blood flow through the graft under color Doppler mode.

### Lower Limb Blood Flow Measurement

Note: Imaging of lower limb blood flow is obtained by Laser Doppler Flow Metry (Moor Instruments, Devon, UK) as previously described [9, 10].

1. Anesthetize the mouse by intraperitoneal injection of 50 mg/kg pentobarbital and assess the depth of anesthesia by pinching the mouse’s feet to ensure the disappearance of foot retraction reaction.
2. Place the mouse in a supine position on the table. Apply the depilatory paste evenly to the skin of the abdomen and lower extremities and wipe up the removed hair with sterile cotton balls.
3. Use a computer-controlled optical scanner to direct a low-power laser beam over the exposed abdomen and lower extremities.
4. Place the scanning head parallel to the exposed site at a distance of about 20 cm.
5. Evaluate and record blood flow perfusion level by the moor FLPIR view V40 software, which is displayed on the video monitor in the form of a color-coded image.

### Plaque Assessment

Note: The recipient mouse is euthanized 1 week after transplantation.

1. Perfuse the pre-transplant and grafted arches according to the donor procedure. For en face oil red staining of the aortic arch, cut the aortic arch longitudinally along the inner curvature and expose the entire intima. Then cut the aortic arch longitudinally along the greater curvature from the proximal end to the left subclavian artery. Fix the aortic arch by using 4% formaldehyde and perform oil red staining for cholesteryl esters content quantification.
2. Transect the aortic arch in the middle, embed the two segments separately into optimal cutting temperature (OCT) medium, and freeze them at − 80 °C immediately.
3. Cut out serial frozen sections of 6 µm thickness on a cryostat.
4. Stain slides by hematoxylin–eosin (H&E) staining for plaque area analysis and oil red staining for cholesteryl esters content quantification.
5. Digitally image all tissues or sections through a microscope and measure the plaque area using Image J software.
6. Use the average of 8–10 sections per aortic arch for statistics.
7. For oil red staining, the percentage of lipid content is calculated by dividing the oil red area by the total plaque area or the total intimal area.

### Statistical Analysis

Data are presented as the mean ± standard error of the mean. The two-tailed, unpaired *t* test is performed using the GraphPad Prism 9 software. Differences with *P* < 0.05 are considered to indicate statistical significance.

## Results

**Fig. 1:**
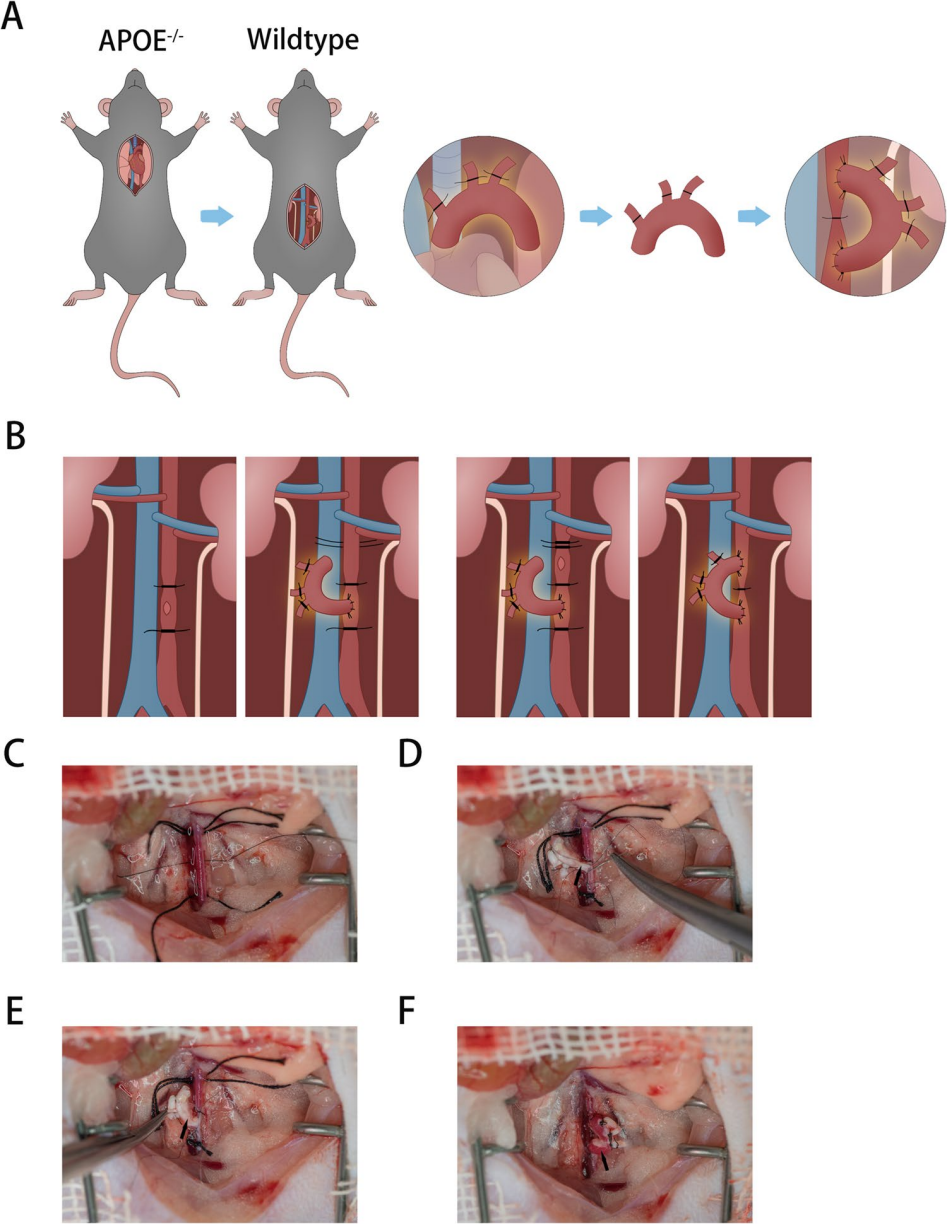
Illustration and scheme of mouse aortic arch transplantation model.** A** Illustration of mouse aortic arch transplantation model; **B** diagrammatic illustration of modified end-to-side anastomotic order;** C** a clear separation of the abdominal aorta and a branch artery; **D** process of anastomosis for distal end of aortic arch; **E** finished anastomosis for distal end of aortic arch; **F** the patency of graft indicated by full of blood flow. **D**–**F** The black arrows denote the aortic arch graft

The illustration of mouse aortic arch transplantation (a segment of aortic arch from ApoE^−/−^ mouse was surgically interposed into an abdominal aorta of a normal wildtype mouse) and the modified anastomosis order from distal to proximal end of abdominal aorta are shown in Fig. 1A and B. According to our modified surgical protocol, it was feasible to reach a clear and successful separation of the abdominal aorta and the branches, smooth side-to-end anastomosis, and unobstructive graft (Fig. 1C–F).

**Fig. 2:**
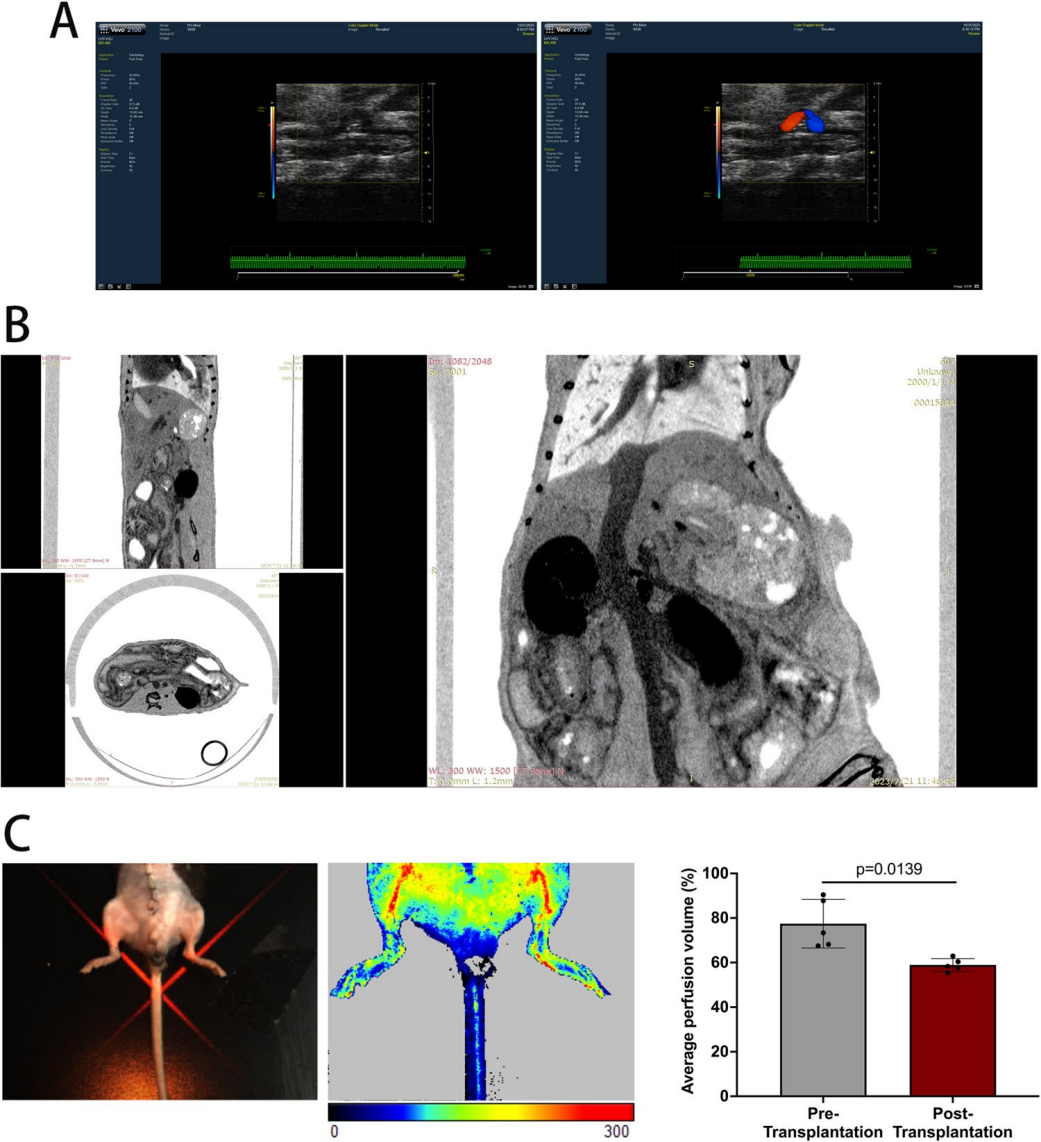
In vivo imaging of aortic arch graft for patency assessment.** A** Two-dimension and Doppler ultrasound imaging showing the aortic arch graft and the blood flow direction;** B** micro-CT imaging of coronal, sagittal, and vertical planes of the aortic arch graft; **C** imaging of lower limb blood flow 1 week after transplantation and its average perfusion volume compared with the pre-transplant group (*N* = 5 in each group), data were analyzed by *t* test; red arrows: abdominal aorta; blue arrows: inferior vena cava; white arrows: aortic arch graft

The blood perfusion in the aortic arch graft was evaluated immediately by observation of the graft pulsation. We also evaluated the patency of graft at 1 week after transplantation by using ultrasound and micro-CT (Fig. 2A and B). Doppler ultrasound imaging showed that blood flow passed through the graft (Fig. 2A), while the contrast agent was successfully perfused from the proximal abdominal aorta through the graft to the distal abdominal aorta in micro-CT imaging (Fig. 2B). We also noted that the blood perfusion in mouse lower limbs at 1 week after transplantation was only slightly decreased compared with the pre-transplant group (58.94 ± 1.28% vs. 77.46 ± 4.88%, *P* = 0.0139) (Fig. 2C), which did not affect the function and daily activities.

**Fig. 3:**
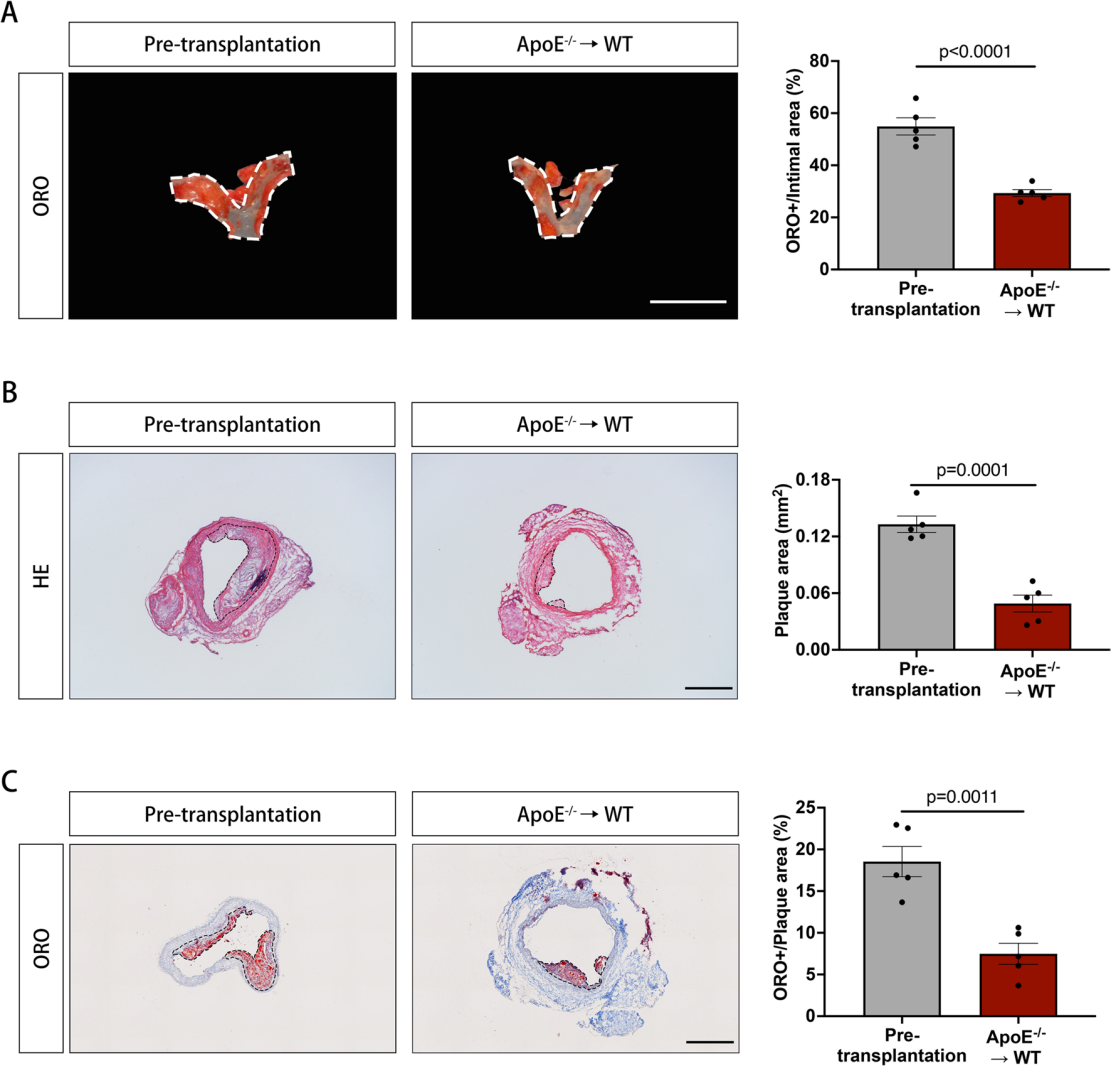
Histologic characteristics of atherosclerotic lesions in aortic arches of ApoE^–/–^ mice before and 1 week after transplantation to wildtype mice. **A** Representative ORO-stained aortic arch grafts and relative lipid content from pre-transplant donors (ApoE^−/−^) and wildtype recipients at 1 week after transplantation;** B** representative H&E-stained sections and quantified plaque area of aortic arch grafts from pre-transplant donors (ApoE^−/−^) and wildtype recipients at 1 week after transplantation; **C** representative ORO-stained sections and relative lipid content of aortic arch grafts from pre-transplant donors (ApoE^−/−^) and wildtype recipients at 1 week after transplantation; HE, hematoxylin–eosin (H&E) staining; ORO, oil red O staining; data were analyzed by *t* test; *N* = 5 in each group; **A** scale bar = 5 mm; **B**, **C** scale bar = 500 µm

For atherosclerosis regression evaluation, the grafted aortic arch segments were removed 1 week after transplantation and lesion size was quantified. In en face staining, it was reduced by approximately 50% in the transplanted grafts compared with the pre-transplant plaque area (29.33 ± 1.34% vs. 54.92 ± 3.29%, *P* < 0.0001) (Fig. 3A). In slides staining, it was reduced by more than 60% in the transplanted grafts compared with the pre-transplant plaque area (0.1329 ± 0.0087 mm^2^ vs. 0.0490 ± 0.0089 mm^2^, *P* = 0.00001) (Fig. 3B). The plaque lipid content was significantly lower in the transplanted grafts than in the pre-transplant group (7.48 ± 1.27% vs.18.54 ± 1.81%, *P* = 0.0011) (Fig. 3C).

## Discussion

Plaque regression is very difficult in patients with atherosclerosis. Lipid-lowering drugs including the powerful inhibitors of proprotein convertase subtilisin kexin-like 9 (PCSK9) could only reduce the mean change in percent atheroma volume of coronary plaque by less than 3% [11], while the plaque regression of grafts rapidly reached to 60% in our aortic arch transplantation mouse model, indicating that this model is a powerful tool to investigate the mechanisms of plaque regression. Atherosclerotic plaque formation usually results from a complex interplay between many factors such as lipid deposition, inflammatory immune reaction, cell migration, and arterial wall injury. However, the mechanisms of plaque regression are largely unknown. Besides to reduce the lipid burden, targets for changing plaque environment should make important contributions to plaque regression. Functional restoration of the endothelium, reversion of the proliferative synthetic vascular smooth cells [12], and mobilization of anti-plaque macrophages [13] have been reported to promote plaque regression. Characterized by dramatically regression of atherosclerotic plaque, the aortic arch transplantation model can be employed to search therapeutic targets for plaque regression.

The mouse atherosclerosis regression model through aortic arch transplantation was first reported by Fisher et al. in 2003 [1]. Unfortunately, few laboratories have employed this model to research plaque regression since then, which may be attributable to the technique difficulty of the transplant surgery and insufficient details of the procedure to facilitate duplication. Therefore, we tried to provide a more detailed, easily reproducible surgical protocol to facilitate the duplication of this model.

Separation of the abdominal aorta from IVC is a basic skill and very important. The purpose of separation is to increase the visual area of the abdominal aorta and expand the operable space for subsequent suture. A key point for this step is to find and ligate the small branches hidden between the aorta and vein to avoid active bleeding, shock, and thrombosis. Different from the thoracoabdominal aortic transplantation model which needs a 4 mm segment separation of abdominal aorta, the aortic arch transplantation model requires a 7 mm or longer segment separation for end-to-side anastomosis. In that case, it is inevitable to separate the abdominal aortic segment below the lumbar artery, where the aorta and vein are tightly held together by transparent connective tissue, while incautious manipulation-induced arteriovenous rupture and bleeding would hardly be stopped. We summarized 4 steps to guarantee the successful separation of the abdominal aorta as follows: starting below the left renal vessels, gently tearing the connective tissue, bluntly dissecting it, injecting saline to increase exposure, threading, and knotting the branches.

How to make abdominal aortotomies is critical for the success rate of arch transplantation. In thoracoabdominal aortic transplantation, transecting the abdominal aorta at the appropriate location can directly obtain two elliptical openings with the trim edge. However, making incisions for aortic arch transplantation requires some microsurgical skills. Here we introduced a method of abdominal aortotomies inspired from mouse heart transplantation [14]. By using a needle of 10–0 nylon suture, we could cut out a section of the anterior aortic wall and the incision presented an ellipse with the trim edge, which greatly reduced the incidence of the incision closure.

Anastomosis is the most critical step of transplantation. The success rate may be improved by modifying the order of proximal to distal anastomosis and anastomosis method. After adjusting the order of anastomosis to distal–proximal, the full arterial segment would facilitate the puncture of the abdominal aorta to avoid damage to the posterior aortic wall. Moreover, the elastic aortic wall was conducive to the puncture of the stitching needle when anastomosis, without tearing the wall due to tension. For traditional anastomosis, stay sutures are placed at the 6 and 12 o’clock positions for the purpose of aligning the edges of the opening and incision to prevent large leakages caused by misalignment, but in this way, the opening and incision are sticking to each other, the exit point of stitching needle is often mistaken and it is easy to run the stitching needle through the left and right cut edge of the opening or incision, causing direct closure of them and occlusion of blood flow. Therefore, we recommended that the stay suture at 6 o’clock position should not be knotted until the left cut edge was closed, by which the space between the opening and the incision was open and the entry and exit point of the stitching needle could be visualized without mistake. What’s more, the running sutures were not pulled tight until the third stitch was finished, which was also based on this consideration.

## Abbreviations

IVC: Inferior vena cava
PCSK9: Proprotein convertase subtilisin kexin-like 9
HE: Hematoxylin–eosin
ORO: Oil red O
VSMCs: Vascular smooth cells
